# Prevalence of Amyotrophic Lateral Sclerosis — United States, 2014

**DOI:** 10.15585/mmwr.mm6707a3

**Published:** 2018-02-23

**Authors:** Paul Mehta, Wendy Kaye, Jaime Raymond, Ruoming Wu, Theodore Larson, Reshma Punjani, Daniel Heller, Jessica Cohen, Tracy Peters, Oleg Muravov, Kevin Horton

**Affiliations:** 1 Division of Toxicology and Human Health Sciences, Agency for Toxic Substances and Disease Registry, CDC.

Amyotrophic lateral sclerosis (ALS), commonly known as Lou Gehrig’s disease, is a progressive and fatal neuromuscular disease; the majority of ALS patients die within 2–5 years of receiving a diagnosis (*1*). Familial ALS, a hereditary form of the disease, accounts for 5%–10% of cases, whereas the remaining sporadic cases have no clearly defined etiology (*1*). ALS affects persons of all races and ethnicities; however, whites, males, non-Hispanics, persons aged >60 years, and those with a family history of ALS are more likely to develop the disease (*1*–*3*). No cure for ALS has yet been identified, and the lack of proven and effective therapeutic interventions is an ongoing challenge. Current treatments available do not cure ALS but have been shown to slow disease progression. Until recently, only one drug (riluzole) was approved to treat ALS; however, in 2017, the Food and Drug Administration approved a second drug, edaravone (*4*). 

This report presents National ALS Registry (Registry) findings regarding ALS prevalence for the period January 1–December 31, 2014, and, for the first time, includes Medicare hospice data and ALS prevalence rates by Census region. ALS prevalence did not change from 2013, remaining at 5.0 cases per 100,000 persons in 2014. Data collected by the Registry are being used to better describe the epidemiology of ALS in the United States and to facilitate research.

In 2008, the U.S. Congress passed the ALS Registry Act, which authorized the creation and maintenance of the Registry by CDC; CDC delegated this responsibility to the Agency for Toxic Substances and Disease Registry (ATSDR) (*5*). The main goals of the Registry are to better describe the incidence and prevalence of ALS, characterize the demographics of persons living with ALS in the United States, and examine potential risk factors such as environmental and occupational influences. Because ALS is not a notifiable disease in the United States, the Registry employs a novel case-finding approach that uses administrative and self-reported data to identify cases, whereas usual noncommunicable disease registries (e.g., cancer) typically rely on data reported from health care providers to identify cases.

ATSDR’s Registry uses a two-pronged approach to identify ALS cases (*6*). The first component applies a pilot-tested algorithm that includes elements such as the *International Classification of Diseases* code for ALS, frequency of visits to a neurologist, and prescription drug use to three large national databases (Medicare, Veterans Health Administration, and Veterans Benefits Administration). The algorithm categorizes cases as “definite ALS,” “possible ALS,” and “not ALS”; only definite ALS cases are entered into the Registry. “Possible ALS” cases are evaluated for conversion to “definite ALS” in subsequent years. The second component comprises a secure web portal to allow persons with ALS to self-register to facilitate identification of cases not collected through the first component (*7*). Cases from both data sources are then merged and deduplicated. In addition, for this report, Medicare hospice data were included for the first time. Once an ALS case is identified, it remains a case until the person is confirmed as deceased by obtaining death data from the National Death Index. The prevalence of ALS was calculated from the Registry by using the deduplicated total number of persons with ALS identified through administrative data and those who self-identified through the portal as the numerator. The 2014 Census estimate was used for the denominator (*8*).

A total of 15,927 persons were identified as having definite ALS across the three national databases and through web portal registration for 2014 (Table). The estimated prevalence for 2014 was 5.0 per 100,000 population, representing no increase from 2013 (5.0 per 100,000). No significant increases were observed across age groups (Figure). The lowest prevalence (0.5 per 100,000 population) was among persons aged 18–39 years, and the highest (20.0) was among persons aged 70–79 years. As in 2013, the prevalence in males (6.3) was higher than that in females (3.6) (Table). The ratio of cases in males to those in females was 1.7:1. The prevalence in whites (5.4) was more than twice that in blacks (2.4).

**TABLE:** Number and percentage of identified cases of amyotrophic lateral sclerosis (N = 15,927) and estimated prevalence, by age group, sex, race, and geographic region — National ALS Registry, United States, 2014

Characteristic	Population*	No. (%) ALS cases	Prevalence estimate (cases per 100,000 population), % (95% CI)
**Age group (yrs)**			
18–39	94,902,312	506 (3.2)	0.5 (0.5–0.6)
40–49	41,479,525	1,587 (10.0)	3.8 (3.5–4.2)
50–59	44,082,258	3,492 (21.9)	7.9 (7.4–8.4)
60–69	33,891,398	4,861 (30.5)	14.3 (13.7–15.0)
70–79	18,995,348	3,807 (23.9)	20.0 (19.2–20.9)
≥80	11,922,597	1,623 (10.2)	13.6 (13.1–14.2)
Unknown	—	51 (0.3)	—
**Sex**			
Males	156,936,487	9,821 (18.6)	6.3 (6.1–6.4)
Females	161,920,569	5,854 (36.8)	3.6 (3.5–3.7)
Unknown	—	252 (1.6)	—
**Race**			
White	233,963,128	12,660 (79.5)	5.4 (5.2–5.5)
Black	40,379,066	988 (6.2)	2.4 (2.3–2.6)
Other	—	863 (5.4)	—
Unknown	—	1,416 (8.9)	—
**U.S. Census region^†^**			
Midwest	67,745,108	3,832 (24.1)	5.7 (5.4–5.9)
Northeast	56,152,333	3,075 (19.3)	5.5 (5.2–5.8)
South	119,771,934	5,682 (35.7)	4.7 (4.6–4.9)
West	75,187,681	3,252 (20.4)	4.3 (4.1–4.5)
Unknown	—	86 (0.5)	—
**Total**	**318,857,056**	**15,927**	**5.0 (4.9–5.1)**

**Abbreviations**: ALS = amyotrophic lateral sclerosis; CI = confidence interval.

* From 2014 U.S. Census data.

^†^ *Northeast:* Connecticut, Maine, Massachusetts, New Hampshire, New Jersey, New York, Pennsylvania, Rhode Island, Vermont; *South:* Alabama, Arkansas, Delaware, District of Columbia, Florida, Georgia, Kentucky, Louisiana, Maryland, Mississippi, North Carolina, Oklahoma, South Carolina, Tennessee, Texas, Virginia, West Virginia; *Midwest:* Iowa, Illinois, Indiana, Kansas, Michigan, Minnesota, Missouri, Nebraska, North Dakota, Ohio, South Dakota, Wisconsin; *West:* Alaska, Arizona, California, Colorado, Hawaii, Idaho, Montana, Nevada, New Mexico, Oregon, Utah, Washington, Wyoming.

**FIGURE:**
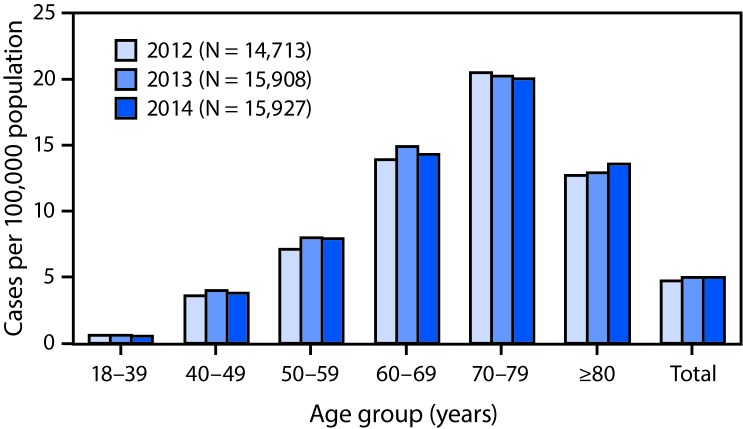
Prevalence of amyotrophic lateral sclerosis (ALS), by age group — National ALS Registry, United States, 2012–2014. **Abbreviation:** ALS = Amyotrophic lateral sclerosis.

Prevalence rates were also calculated for the four U.S. Census regions: Northeast, South, Midwest, and West. Rates were highest in the Midwest (5.7 per 100,000 population), followed by the Northeast (5.5), the South (4.7), and the West (4.3) (Table).

## Discussion

Data sources for the Registry remain unchanged, but the national administrative data now include hospice data from Medicare. The Registry’s novel approach of using national administrative databases is the cornerstone for identifying ALS cases because most of the definite ALS cases from 2010 to 2014 originate from this source.

Since publication of the first surveillance summary that reported analyzed data for 2010–2011 (*2*) and for subsequent years (*3*), ALS has remained more prevalent in whites, males, and persons aged ≥60 years; current patterns are similar to those identified during 2010–2013. These patterns remain unchanged for 2014. It was hypothesized that the prevalence would increase in 2014 with the additional hospice data; however, this was not the case. Additional years of data are needed to determine whether ALS cases are increasing, decreasing, or remaining the same in the United States. The inclusion of Medicare hospice data for the first time in 2014 did not affect estimated ALS prevalence. Many patients identified through hospice data had been previously identified in either Medicare data, Veterans Health Administration data, Veterans Benefits Administration data, or the web portal. The Registry continues to evaluate additional data sources for case identification as well as ways to increase self-registration through the secure web portal to increase case ascertainment.

Prevalence rates by U.S. Census regions are consistent with ALS demographics. Overall, whites have a higher prevalence of ALS than blacks. The higher ALS prevalence in the Midwest and Northeast likely reflects the higher proportion of whites, compared with the South and West (*8*). The lowest prevalence in the West Census region is most likely related to the population diversity in states such as California (*8*).

The Registry continues to expand ALS research nationally. In January 2017, the National ALS Biorepository (Biorepository), a component of the Registry, was launched. The Biorepository is novel in several ways. First, it obtains samples from Registry enrollees via in-home collection (e.g., blood, hair, or saliva) and postmortem collection (e.g., brain, bone, spinal cord, cerebrospinal fluid, muscle, and skin) at no charge to patients or their caregivers. Currently, the few existing ALS biorepositories largely have samples from specific clinics or medical practices, and the samples that are left over from previous clinical trials in the United States. Second, specimens from the National ALS Biorepository are collected from a geographically representative sample of Registry enrollees. The sample of persons recruited to participate in the Biorepository correlates with the population distribution of the United States and each year will include at least one person from each state. Third, these deidentified samples are paired with completed risk factor survey data (e.g., occupational and military history) from the Registry. Researchers are currently able to request samples alone or paired with risk factor data. The availability of additional specimens from a national sample of ALS patients further expands research potential on the genetics, potential biomarkers, environmental pollutants, and etiology for ALS. Additional information for requesting samples and/or risk factor data is available at https://wwwn.cdc.gov/als/ALSRegistryResearchApplicationInfo.aspx.

The findings in this report are subject to at least four limitations. First, ALS is not a notifiable disease, and ensuring that all newly diagnosed and prevalent ALS cases in the United States are collected in the Registry is challenging; therefore, the possibility of underascertainment exists. Second, although every attempt was made to deduplicate the files, differences in fields collected by the different sources, misspellings of names, and data entry errors could have prevented records from merging correctly. However, it is unlikely that this occurred in numbers sufficient to affect the overall conclusions. Third, the calculation of ALS incidence with Registry data is not possible at this time because the date of diagnosis is not collected through the large administrative database approach, and cases without a date of diagnosis account for more than two thirds (68%) of cases in the Registry. Finally, the Registry has been officially active since October 2009 and is still being enhanced. As more persons with ALS enroll and complete surveys, a better understanding of possible risk factors might emerge (*2*,*3*).

Establishment of the National ALS Registry, as well as the newly launched National ALS Biorepository, fills a critical scientific gap by providing estimates of prevalence of this disease and facilitates further study of risk factors and etiology. The National ALS Registry continues to be improved and enhanced, increasing its potential for ALS research and detection of more ALS cases. ATSDR is committed to advancing ALS research and monitoring trends of ALS prevalence in the United States.

**Summary**

**What is already known about this topic?**

Amyotrophic lateral sclerosis (ALS), commonly known as Lou Gehrig’s disease, is a progressive and fatal neuromuscular disease. Familial ALS, a hereditary form of the disease, accounts for 5%–10% of cases; the remaining sporadic cases have no clearly defined etiology.

**What is added by this report?**

A total of 15,927 persons were identified as having definite ALS across three national databases (Medicare, Veterans Health Administration, and Veterans Benefits Administration) and through web portal registration for 2014. The estimated ALS prevalence for 2014 was 5.0 cases per 100,000 population, the same as 2013 estimate.

**What are the implications for public health practice?**

Through ongoing enhancements and expanded outreach and promotion, the National ALS Registry has the potential to expand ALS research and detect more ALS cases in the United States.
